# Comparison of Survival between Single-Access and Conventional Laparoscopic Surgery in Rectal Cancer

**DOI:** 10.1155/2021/6684527

**Published:** 2021-03-17

**Authors:** Siripong Sirikurnpiboon

**Affiliations:** ^1^Division of Colorectal Surgery, Department of Surgery, Rajavithi Hospital, Bangkok, Thailand; ^2^College of Medicine, Rangsit University, 2, Phyathai, Rachathewi District, Bangkok 10400, Thailand

## Abstract

**Introduction:**

Innovative laparoscopic surgery for rectal cancer can be classified into 2 types: firstly, new instruments such as robotic surgery and secondly, new technique such as single-access laparoscopic surgery (SALS) and transanal total mesorectal excision (TaTME). Most reports of SALS for rectal cancer have shown pathologic outcomes comparable to those of conventional laparoscopic surgery (CLS); however, SALS is considered to be superior to CLS in terms of lower levels of discomfort and faster recovery rates. This study aimed to compare the survival outcomes of the two approaches.

**Methods:**

From 2011 to 2014, 84 cases of adenocarcinoma of the rectum and anal canal were enrolled. The operations were anterior, low anterior, intersphincteric, and abdominoperineal resections. Data collected included postoperative outcomes. The oncological outcomes recorded included 3-year and 5-year survival, local recurrence, and metastasis.

**Results:**

SALS was performed on 41 patients, and CLS was utilized in 43 cases. The demographic data of the two groups were similar. Intraoperative volumes of blood loss and conversion rates were similar, but operative time was longer in the SALS group. There were no significant differences in postoperative complications or pathological outcomes. The oncologic results were similar in terms of 3-year survival (100% and 97.7%; *p* = 1.00), 5-year survival (78.0% and 86.0%; *p* = 0.401), local recurrence rates (19.5% vs 11.6%, *p* = 0.376), and metastasis rates (19.5% vs 11.6%; *p* = 0.376) for SALS and CLS, respectively.

**Conclusion:**

SALS and CLS for rectal and anal cancer had comparable pathological and survival results, but SALS showed some superior benefits in the early postoperative period.

## 1. Introduction

Laparoscopic surgery is a generally accepted surgical technique for colorectal cancer surgery and is superior to the open approach in some outcomes such as faster postoperative recovery, shorter length of hospital stay (LHS), and potentially reduced postoperative mortality [1–5]. With regard to rectal cancer, several studies have demonstrated that laparoscopic surgery is also safe and effective and does not compromise oncological outcomes; thereby, it is often recommended as an alternative method to open surgery in many international guidelines [6–10]. Single-access laparoscopic surgery (SALS) is a surgical technique that was developed to improve upon multiport conventional laparoscopic surgery (CLS) by limiting incisions and alleviating pain. The concept lies in the use of a single multichannel port site to perform the entire procedure; however, in rectal cancer, there are some drawbacks, such as instrument collision, reduced ability to create a working space in narrow pelvises, and also endostaple application problems. The current author has previously reported some of the techniques used to facilitate this type of surgery in rectal cancer and its short-term outcomes [11–13]; however, the long-term oncological results are still debated. In this study, we report the long-term oncological outcomes of SALS for rectal and anal adenocarcinoma.

## 2. Materials and Methods

This was a retrospective study of minimally invasive operations for rectal cancer performed between January 2011 and January 2014. Ninety-one patients with a preoperative diagnosis of adenocarcinoma of the rectum or anal canal confirmed by pathological diagnosis were enrolled, but neoadjuvant treatment was not provided in either group as the patients refused to give consent for radiation. In the preoperative period, four patients were excluded from the study: one refused laparoscopic surgery; one required an emergency operation for colonic obstruction; and two were unable to undergo the operation because of severe medical diseases. In the intraoperative period, three patients were excluded because they had to convert to laparotomy owing to severe adhesion. Of the 84 remaining patients, SALS was performed in 41 cases and CLS in the other 43. Operative techniques employed were as described in the author's previous studies. [11–13]. The protective ileostomy was created at right lower quadrant area, and drain was placed by adding incision at left lower quadrant area.

Data were collected in 4 stages: first, preoperative demographic data were collated together with details of type of operation (Table 1); second, intraoperative and early postoperative data such as operative time, blood loss volume, complications, pain score using the visual analog scale, wound length (SALS group only), hospital stay, and conversion rate were recorded (Table 2); third, pathological outcomes including stage, mesorectal grade, and surgical margin were reported (Table 3); and lastly, oncological outcomes were analyzed.

## 3. Statistical Analysis

SPSS version 17.0 (SPSS Inc. Chicago, IL, USA) was used to analyze the data. A univariate comparison of categorical data was analyzed by *χ*^2^ or Fisher's exact test. Mann–Whitney *U* test or *t*-test was used to compare quantitative variables. The Kaplan–Meier test was used for survival analysis.

## 4. Results

Total colorectal cancer surgery was performed on 516 patients in study period. A total of 84 patients were enrolled in this study, with 41 in the SALS group and 43 in the CLS group. Median follow-up was 74 (30–100) months in SALS and 64 (18–96) months in CLS group (*p* = 0.128). Of the 41 cases in the SALS group, 19 were male and 22 were female, with the mean ± SD age of 63.97 ± 13.05 years and mean BMI ± SD of 22.20 ± 4.00 kg/m^2^. With regard to ASA classification, class II, with 28 patients (68.3%), was the most common. The CLS group had nearly the same male-to-female ratio (20 : 23) with a mean age of 61.74 ± 12.03 years and a mean BMI of 23.14 ± 2.89 kg/m^2^. ASA class II was the most prevalent with 31 patients (72.1%). No difference was found in clinical staging. The imaging study was MRI and CT with endoscopic ultrasonography. The demographic data of the two groups were comparable except that there were more anal canal and lower rectal cancer cases in the SALS group (*p* = 0.052), eventually resulting in more APR procedures in these patients (*p* = 0.058) but not statistically significant, as displayed in Table 1.

Intraoperative data revealed longer operative times in the SALS patients than in their CLS counterparts (268.29 ± 50.73 minutes vs. 233 ± 53.80 minutes, *p* = 0.003), but all other factors such as volume of blood loss and conversion to laparotomy rates were comparable. With regard to postoperative data, complication rates were similar in the two groups. There were some advantages with respect to short-term outcomes in the SALS group, including reduced pain, earlier recovery of bowel movement, and shorter hospital stay, and these differences were statistically significant. Intraoperative and postoperative data are shown in Table 2.

Pathologic results showed that pathologic staging and number of nodes harvested were comparable in the two groups; however, there were some differences in *T* stage, with more advanced stage in the CLS group, and these differences were statistically significant (*p* = 0.016). Specimen quality defined by mesorectal grade showed similar rates of complete mesorectal specimen in the two groups (73.2% vs 60.5%, *p* = 0.188), and all patients had negative CRM and distal rectal margin. Pathological results are shown in Table 3.

**Figure 1:**
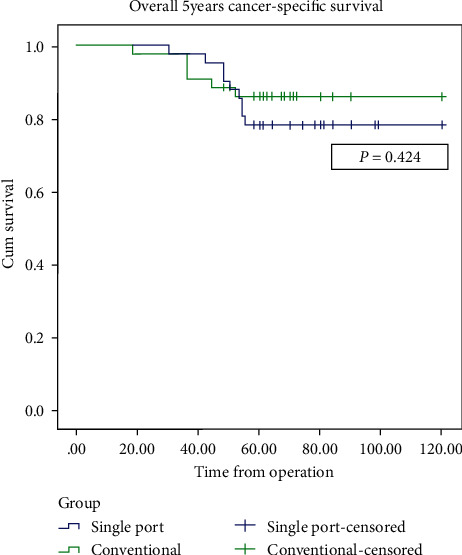
Kaplan–Meier curve of overall cancer survival.

**Figure 2:**
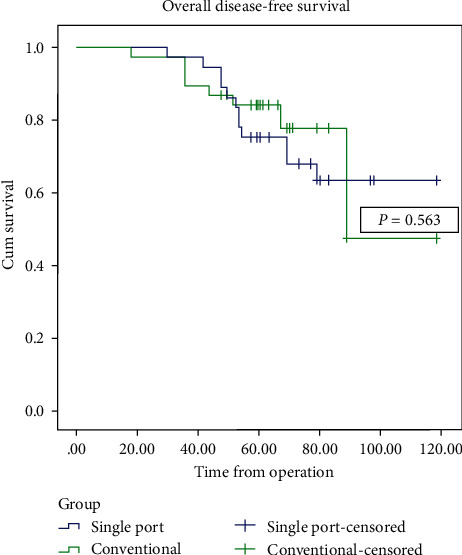
Kaplan–Meier curve of disease free survival.

With regard to primary results, all oncological outcomes were similar in the two groups. The 3-year survival rates were 100% and 97.7% in the SALS and CLS groups, respectively (*p* = 1.00), while 5-year survival was 78.0% and 86.0%, respectively (*p* = 0.401). Local recurrence rates were 19.5% in the SALS group with an average time to recurrence of 29.75 ± 9.8 months compared with 11.6% in the CLS group with an average time to recurrence of 19.60 ± 9.83 months (*p* = 0.105). In the SALS group, total local recurrence patients were 7 patients but 1 of all was inoperable. In CLS groups, total local recurrence was 4 patients but 2 were inoperable. Distant metastatic rates were 19.5% and 11.6% in the SALS and CLS groups, respectively (*p* = 0.376), with average time to metastasis of 43.37 ± 6.52 and 40.60 ± 13.22 months, respectively (*p* = 0.679). In the SALS group, metastasis site was liver 4 patients, lung 3 patients, and combined lung and liver 1 patient. In the CLS group, metastasis site was lung 4 patients and liver 1 patient (*p* = 0.361). The Kaplan–Meier survival curves for overall cancer survival (OS) and disease free survival (DFS) are shown in Figures 1 and 2.

At univariate analysis, DFS was affected with *T* pathological stage (*p* = 0.15), pathological stage (*p* = 0.025), presence of LVI (p-0.008), and CRM distance 1–3 mm (*p* < 0.005). OS was affected with pathological stage (*p* = 0.047), presence of LVI (*p*-0.003), and CRM distance 1–3 mm (*p* < 0.005).

## 5. Discussion

The results of this study showed that rectal cancer operations can be performed by SALS without compromising oncological outcomes. SALS can also offer some additional short-term advantages over conventional multiport surgery, including lower postoperative pain, earlier bowel movement, and decreased length of hospital stay.

Single-access laparoscopic surgery is referred to a variety of names, such as single-incision laparoscopic surgery (SILS) and single-port laparoscopic surgery. The first single-access laparoscopic colonic resection, a right hemicolectomy, was reported by Remzi et al. [14]. Subsequently, the first colonic SILS case series was reported of six right-sided and one left-sided resection with good short-term outcomes [15]. Since then, SALS for colonic surgery has been evaluated in multiple studies, including RCT and meta-analyses, revealing that it yields some benefits in the postoperative period and in terms of cosmetic results, in comparison to CLS [16–20]. For rectal cancer surgery, however, some difficulties persist in performing total mesorectal excision (TME) using a single-port platform, and these are mostly due to instrument collision, problems in creating traction, and difficulties with stapler firing. These problems may lead to below-average specimen quality, especially with respect to mesorectal completeness and circumferential margin, and they may eventually lead to less satisfactory oncological outcomes [21–25]. This study showed that, with meticulous care, these problems can be avoided, and previous research has shown comparable pathological outcomes between the two modalities [12]. In this study, there were no differences in rates of complete mesorectal incision, circumferential margin, or distal margin in the specimens from the SALS and CLS subjects.

Regarding primary outcomes, the two groups had comparable oncological results, including 5-year overall survival, local recurrence, and metastatic rate; even though, compared with the published literature, this study had higher local recurrence rates (LR) at 11–15%, [26–28], and this may be explained by the fact that none of the patients in this study had neoadjuvant therapy because the patients who received neoadjuvant in same period had local recurrence rate of 8%. A study by Gash KM et al. reported a 4.9% local recurrence rate using the single-port technique after neoadjuvant therapy; [29] however, the 13% LR rate in postoperative radiotherapy (RT) found in the study by Saur et al. was reasonably similar to the LR in this series [27]. Other factors often considered to potentially increase the risk of recurrence, such as male sex and high BMI, which were not significantly different in the two groups in this study.

With regard to survival, this study found that 5-year survival rates were comparable in the two groups and were similar to those found in other previous reports of conventional laparoscopic rectal surgery [30–33]. Factors related to 5-year survival included circumferential margin, number (CRM), harvested lymph node, mesorectal grade, and postoperative complications [34]. CRM was first identified as a significant factor in survival from rectal cancer by Quirke et al. [35]. In later studies, CRM involvement was found to increase the risk of local recurrence and distant metastases and to adversely affect survival rates [36, 37]. The distance of CRM has a correlation with survival and local recurrence [38] and recommendation is 2 mm [39]. This study showed result in the same correlation but limitation did not record the location of tumor for correlation with CRM. However, some research studies have shown slightly different results. A study by Hall et al. [40] reported that although circumferential margin positive was associated with poor prognosis in terms of distant metastasis and survival in rectal cancer, it did not increase the local recurrence rate owing to the early progression of distant metastasis rather than local failure [41]. Another study of patients who underwent neoadjuvant therapy showed that CRM had no effect on local recurrence or survival. A study from Sauer et al. revealed that preoperative chemo radiotherapy did not seem to be more effective than postoperative chemo radiotherapy, and this is in agreement with the findings of the previous study [42]. It is therefore debatable whether or not neoadjuvant therapy is more beneficial than local control. In this study, we were able to achieve CRM negative in all patients in both groups, and this may be one of the factors associated with good survival.

Harvested lymph node, the lymph node metastases in colon and rectal cancer, is recognized as one of the important prognostic factors for long-term outcomes, and the presence of lymph node involvement is an indicator for adjuvant treatment. The optimal number of harvested lymph nodes to be examined is another point of discussion. Most guidelines based on these studies [43–45] recommend examination of 12 lymph nodes; however, in clinical practice, most surgeons try to harvest as many lymph nodes as possible although limitations are frequent because of patient demographics, tumor location, neoadjuvant, and tumor biology [46]. With regard to lymph node ratio (LNR) which is defined as the ratio between positive lymph nodes and total number harvested, some studies [47] have shown that overall 5-year survival decreased in a ratio near to 1.00. Currently, LNR is still not incorporated into universal staging. In this study, the average number of harvested lymph nodes was more than 12 in both groups, possibly due to the fact that all patients refused neoadjuvant therapy.

Mesorectal grade is one indication of completeness of surgery and has an effect on oncologic results. Maslekar et al. [48] reported local recurrence in terms of quality of mesorectal grade. Grade 1 had 41% recurrence rate, grade 2 had 5.7%, and grade 3 had 1.6%. Although Jeyarajah et al. [49] failed to reveal a correlation between local recurrence and mesorectal grade, they found that pCRM-negative status could be more likely to have an effect on survival than mesorectal grade. Another recent study confirmed that CRM had a more significant effect on survival than mesorectal grade [22] even when neoadjuvant therapy was used.

Achieving negative CRM and good mesorectal grade requires adequate preoperative planning and proper intraoperative laparoscopic instruments and techniques. In this study, SALS had a longer operative time than CLS, which is to be expected, as SALS requires more time to cover instrument handling, with slow movement to minimize tissue dissection.

The short-term benefits of SALS over CLS were clear in the immediate postoperative period in the form of significantly lower postoperative pain scores, shorter time to first bowel movement, and reduced length of hospital stay [12, 50].

With regard to long-term complications with SALS, in this study, there were no clinical incision hernias but CT during surveillance found one third incision hernia in umbilical wound, and none of them needed surgical correction, and wound length was around 5 cm, and this could be an advantage for patients who worry about cosmetic results. In a meta-analysis study comparing SALS and CLS, the odds of the incidence of incisional hernia after SALS was 2.83 times greater than after CLS, but the need for surgical correction of incisional hernia was not significantly different [51]. The risk factors of incisional hernia such as greater age, male sex, incision for specimen extraction, and BMI > 30 [52–54] highly varied, and only high BMI is generally agreed. In this study, the average BMI was lower than the risk level (22.05 ± 4.03 kg/m^2^).

There were several limitations to this study. Firstly, it was retrospective, and there were no strong selection criteria in determining which patient underwent which operation. Second, the neoadjuvant in study period is not routine after MDT meeting and consent. The patients were able to choose the means of treatment by themselves. Third, the wound length in the CLS group was not recorded in postoperative period. Also, there was insufficient monitoring of postoperative pain control and wound cosmesis satisfaction.

Single-access laparoscopic surgery and conventional laparoscopic surgery for rectal and anal cancer had comparable pathological and survival results although single-access laparoscopic surgery yielded some short-term advantages over CLS in the postoperative period in some patients.

## Figures and Tables

**Table 1:** Demographic data.

	SALS (*n* = 41)	CLS (*n* = 43)	*p*
Age (years) (mean ± SD.)	63.97 ± 13.05	61.74 ± 12.03	0.417
Sex (M : F)	19 : 22	20 : 23	0.939
BMI (kg/m^2^) (mean ± SD.)	22.20 ± 4.00	23.14 ± 2.89	0.222

ASA classification (%)			
I	7 (18.4)	9 (20.9)	0.331
II	28 (68.3)	31 (72.1)	
III	6 (14.6)	3 (7.0)	

Location (%)			
Anal canal	18 (43.9)	12 (27.9)	0.052
Lower rectum	12 (29.3)	9 (20.9)	
Mid rectum	9 (22.0)	20 (46.5)	
Upper rectum	2 (4.9)	2 (4.7)	

Imaging *T* stage (%)			
II	10 (24.4)	31 (75.6)	0.407
III	14 (32.6)	29 (67.4)	

Imaging *N* stage (%)			
0	6 (14.6)	9 (20.9)	0.621
I	8 (19.5)	10 (23.3)	
II	27 (65.9)	24 (55.8)	

Clinical stage (%)			
I	0 (0.0)	1 (2.3)	0.168
II	6 (14.6)	10 (23.3)	
III	35 (85.4)	32 (74.4)	

Operation (%)			
Abdominoperineal resection	27 (65.9)	18 (41.9)	0.058
Low anterior resection	12 (29.3)	23 (53.5)	
Anterior resection	2 (4.9)	1 (2.3)	
Intersphincteric resection	0 (0.0)	1 (2.3)	

SD: standard deviation; ASA classification: Anesthesia Society of America Physical Classification.

**Table 2:** Intraoperative and postoperative data.

	SALS (*n* = 41)	CLS (*n* = 43)	*p*
Operative time (mean ± SD) (minutes)	268.29 ± 50.73	233 ± 53.80	**0.003** *∗*
Blood loss (mean ± SD.) (ml)	196.34 ± 89.01	215.1 ± 88.99	0.337
First bowel movement (mean ± SD.) (days)	2.78 ± 0.61	3.18 ± 0.87	**0.016** *∗*
Hospital stay (mean ± SD.) (days)	6.8 ± 1.10	8.3 ± 2.88	**0.003** *∗*
Wound length (mean ± SD.) (cm)	4.8 ± 1.0	N/A	N/A
Pain score 24 hrs (mean ± SD.) (VAS)	4.65 ± 0.99	5.48 ± 0.82	**<0.001** *∗*
Pain score 48 hrs (mean ± SD.) (VAS)	3.63 ± 0.82	4.23 ± 1.15	**0.008** *∗*
Complications (%)			0.527
None	34 (82.9)	35 (83.3)	
Lung	3 (7.3)	4 (9.5)	
Cardio	1 (2.4)	0 (0.0)	
SSI			
Abdomen	0 (0.0)	1 (2.4)	
Perineum	0 (0.0)	1 (2.4)	
Anastomotic leakage	1 (2.7)	0 (0.0)	

SSI: surgical site infection; VAS: visual analog scale; CM: centimeter; ML: milliliter.

**Table 3:** Pathologic data.

	SALS (*n* = 41)	CLS (*n* = 43)	*p*
Pathological stage (%)			0.920
I	1 (2.4)	2 (4.7)	
II	14 (34.1)	14 (32.6)	
III	26 (63.4)	27 (62.8)	
Mesorectal grade (%)	0 (0.0)	0 (0.0)	0.188
1	11 (26.8)	17 (39.5)	
2	30 (73.2)	26 (60.5)	
3			
CRM negative	41 (100.0)	43 (100.0)	1.00
CRM distance (%)	19 (46.3)	20 (46.5)	0.388
1–2 mm	19 (46.3)	14 (32.6)	
2.1–5 mm	3 (7.3)	9 (20.9)	
>5 mm			
Distal margin (median, min-max) (mm)	20 (7–60)	20 (5–50)	0.747
LN achieved (mean ± SD)	14.63 ± 5.09	15.53 ± 5.76	0.900
LVI (%)	15 (36.6)	17 (39.5)	0.825
PNI (%)	7 (16.3)	7 (17.1)	1.00

CRM: circumferential margin; MM: millimeter; LVI: lymphovascular invasion; PNI: perineural invasion.

## Data Availability

The data used to support the findings of this study are available from the corresponding author upon request.
